# Discrete adipose-derived stem cell subpopulations may display differential functionality after in vitro expansion despite convergence to a common phenotype distribution

**DOI:** 10.1186/s13287-016-0435-8

**Published:** 2016-12-01

**Authors:** Frederik Mølgaard Nielsen, Simone Elkjær Riis, Jens Isak Andersen, Raphaëlle Lesage, Trine Fink, Cristian Pablo Pennisi, Vladimir Zachar

**Affiliations:** 1 Laboratory for Stem Cell Research, Department for Health Science and Technology, Aalborg University, Aalborg, Denmark; 2 Department of Bioengineering, Polytech Nice-Sophia Engineering School, Nice, France

**Keywords:** Adipose-derived stem cells, Subpopulations, FACS, Polychromatic cell sorting, Phenotype, Antigenic repertoire, Scratch assay

## Abstract

**Background:**

Complex immunophenotypic repertoires defining discrete adipose-derived stem cell (ASC) subpopulations may hold a key toward identifying predictors of clinical utility. To this end, we sorted out of the freshly established ASCs four subpopulations (SPs) according to a specific pattern of co-expression of six surface markers, the CD34, CD73, CD90, CD105, CD146, and CD271, using polychromatic flow cytometry.

**Method:**

Using flow cytometry-associated cell sorting and analysis, gating parameters were set to select for a CD73^+^CD90^+^CD105^+^ phenotype plus one of the four following combinations, CD34^−^CD146^−^CD271^−^ (SP1), CD34^−^CD146^+^CD271^−^ (SP2), CD34^+^CD146^+^CD271^−^ (SP3), and CD34^−^CD146^+^CD271^+^ (SP4). The SPs were expanded 700- to 1000-fold, and their surface repertoire, trilineage differentiation, and clonogenic potential, and the capacity to support wound healing were assayed.

**Results:**

Upon culturing, the co-expression of major epitopes, the CD73, CD90, and CD105 was maintained, while regarding the minor markers, all SPs reverted to resemble the pre-sorted population with CD34^−^CD146^−^CD271^−^ and CD34^−^CD146^+^CD271^−^ representing the most prevalent combinations, followed by less frequent CD34^+^CD146^−^CD271^−^ and CD34^+^CD146^+^CD271^−^ variants. There was no difference in the efficiency of adipo-, osteo-, or chondrogenesis by cytochemistry and real-time RT-PCR or the CFU capacity between the individual SPs, however, the SP2^CD73+90+105+34-146+271-^ outperformed others in terms of wound healing.

**Conclusions:**

Our study shows that ASCs upon culturing inherently maintain a stable distribution of immunophenotype variants, which may potentially disguise specific functional properties of particular downstream lines. Furthermore, the outlined approach suggests a paradigm whereby discrete subpopulations could be identified to provide for a therapeutically most relevant cell product.

**Electronic supplementary material:**

The online version of this article (doi:10.1186/s13287-016-0435-8) contains supplementary material, which is available to authorized users.

## Background

Since the seminal discovery as cells being capable of trilineage commitment [1], the adipose-derived stem cells (ASCs) have come into the spotlight of cell-based and regenerative approaches. There is ever-increasing number of clinical applications, but among the most significant myocardial infarction [2], critical limb ischemia [3], cytoprotection after radiation-induced tissue damage [4], attenuation of acute kidney injury [5], and wound healing [6] can be listed. From the point of regenerative effect, however, it is not their differentiation potential, rather the immunomodulatory, pro-angiogenic, and anti-apoptotic properties [7–10] that play a role.

The immunophenotypical delineation of ASCs originally implicated expression of three hallmark epitopes, the CD73, CD90, and CD105 [11–13]. They were found expressed to a high degree, minimally around 60%, but not completely [14–16], thus leaving open a question of the significance of subpopulations with different surface repertoire. In the face of wide acceptance of this basic profile, attempts have been made to identify additional markers that would be associated with ASC stemness. Apart from the intracellular molecules, which are less suitable for prospective analysis of live subpopulations, such as Sox2 [17], Nanog [18], a whole array of surface markers, including CD117 [19], CD24, CD34, CD146, CD271 [20–23], CD133, CD200, CD362, Stro-1 [24], and SSEA4 [25] have been reported. Out of these molecules the CD34, CD146, and CD271 appeared to correlate most consistently with physiological superiority or higher plasticity [8, 11, 22, 23, 26–33]. The ASCs thus represent a heterogenous mixture of stem and progenitor cells, the composition and properties of which also tends to change during in vitro growth. It is plausible that some discrete subpopulations render specific therapeutic effect better than others. Hence, better understanding of the relationship between immunophenotype and function would be instrumental in advancing the clinical utility of ASCs.

Previous research highlighted the difficulty of inferring the therapeutic potential for a specific application when looking just at a few surface epitopes. As a result, a more recent recommendation underscores examining directly the functional properties using appropriate in vitro surrogate testing for the particular in vivo effect, in order for dedicated batches of ASCs to become a valid therapeutic product [34]. Since the establishment of surface markers as clinical predictors would be highly beneficial, we propose an approach whereby a fine delineation of discrete subpopulations on the basis of more complex co-expression repertoire of multiple markers would be undertaken. To this end, we set out to investigate four of the most prevalent subpopulations based on a polychromatic staining protocol consisting of six selected markers, including CD34, CD73, CDC90, CD105, CD146, and CD271. The FACS purified cells were subsequently grown and analyzed in cloning, differentiation, and wound-healing assays.

## Methods

### ASC isolation and culture

The stromal vascular fraction (SVF) was prepared from abdominal subcutaneous fat of a male donor (44 years, BMI 25.4) according to a procedure described previously by our laboratory [35]. Before donation, a written informed consent was obtained, and the protocol was approved by the regional committee on biomedical research ethics of Northern Jutland, Denmark (project no. VN 2005/54). This study complied with the principles of the Declaration of Helsinki.

**Fig. 1:**
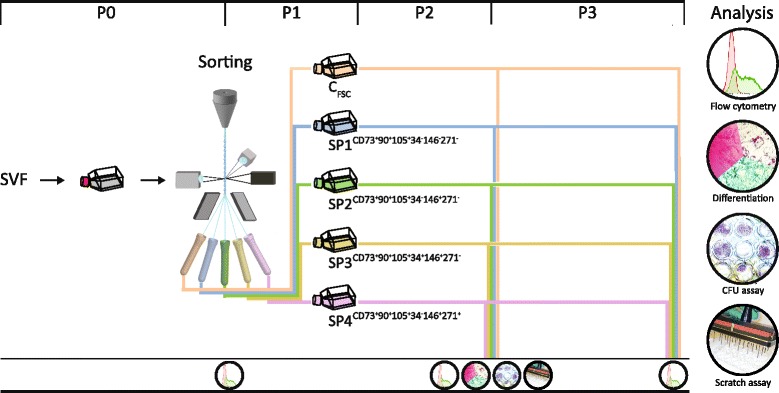
Scheme of the experimental setup. SVF harvested from a single donor was initially seeded out and cultured until reaching confluency (P0). The ASCs were at this point sorted out into a control group (C_FCS_) or four subpopulations (SP1–4) based on a forward- vs. side-scatter (FSC/SSC) gate or patterns of CD markers co-expression, respectively. All sorted populations were subsequently analyzed for immunophenotype at passages P0, P2, and P3, and adipo-, osteo-, and chondrogenic differentiation, clonogenic potential, and wound healing capacity at passage P2. The entire experiment was performed twice independently. *Abbreviations*: *SVF* stromal vascular fraction, *C* control, *SP* subpopulation, *FSC* forward scatter, *CFU* colony-forming unit

The suspension was seeded sparsely and grown in α-Minimum Essential Medium with GlutaMAX (A-MEM) supplemented with 10% fetal calf serum (FCS) and antibiotics (all from Invitrogen, Taastrup, Denmark) for 5 days before sorting (P0). After the sorting, the cultures were established at a density of 500 cells/cm^2^ and allowed to reach a confluency of 80% (P1). At this point, the cells were passaged using TrypLE (Invitrogen), and reseeded at a density of 1000 cells/cm^2^ (P2). Cells were grown under identical conditions during passage three (P3). Specific investigations were performed at the end of passages two and three as indicated in Fig. 1.

### Flow cytometric analysis and cell sorting

The viability and six surface epitopes were analyzed simultaneously on the trypsinized cells using the Live/Dead Fixable Aqua Dead Cell Stain Kit (Molecular Probes, Taastrup, Denmark) and a batch of directly labeled antibodies (all from BD Biosciences, Albertslund, Denmark), respectively (Table 1). The cells were incubated first with the viability reagent for 20 min, followed by a mixture of antibodies optimally diluted in phosphate-buffered saline with 0.1% BSA and 0.25 mM HEPES for 30 min. The working dilutions were based on previous titrations. All incubations and washings were done at 5 °C in the dark. In addition to experiment samples, samples each missing one of the antibodies were prepared to provide for fluorescence minus one (FMO) controls.

**Table 1:** Reagents used for flow cytometric analysis and sorting

Reagent	Fluorochrome	Ex-Em (nm)
Live/Dead	Aqua Blue	355-530/11
anti-CD34	BUV395	355-395/25
anti-CD73	FITC	488-513/26
anti-CD90	PerCP-Cy5.5	488-710/45
anti-CD105	APC	640-664/22
anti-CD146	PE-CF594	561-614/20
anti-CD271	PE-Cy7	561-795/70

*Ex* excitation wavelength, *Em* emission window

**Fig. 2:**
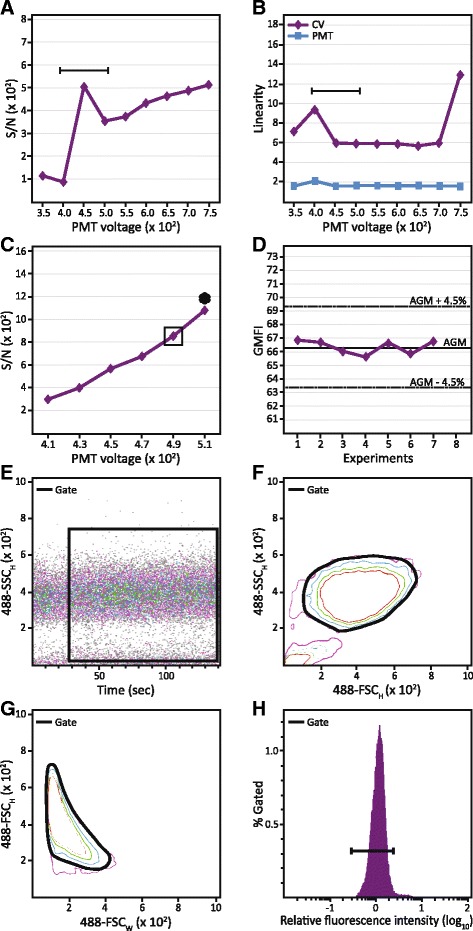
Flow cytometric and gating strategy using the E_x_640 - E_m_664/22 nm channel. **a** Spherotech 6 peak Rainbow beads were used to identify a useful range of PMT potential. The bracket indicates voltage investigated during ensuing optimization. **b** PMT linearity and CV from the same experiment. **c** The narrowed range of PMT voltages was analyzed for optimal S/N using BD Biosciences Compensation beads conjugated with the labeled antibodies used in this study. The voltage that produced the highest S/N with no apparent double negativity was then chosen for each channel and its respective antibody (*in box*). ^*^Double negativity. **d** To ensure the reproducibility of the experimental setup, the Spherotech single peak Rainbow beads were analyzed for 5000 events in each channel every time an experiment was conducted. **e** For sorting, the cells were first gated using SSC_Height_ vs. time plot to control for the stability of the flow cytometer. **f** The noise and debris were removed using FSC_Height_ vs. SSC_Height_ contour plot. **g** The doublets were discriminated and removed using FSC_Width_ vs. FSC_Height_ contour plot. **h** The live cells were identified by means of a viability stain. *Abbreviations*: *PMT* photomultiplier, *CV* coefficient of variation, *S/N* signal-to-noise ratio, *AGM* average geometric mean, *GMFI* geometric mean of fluorescence intensity, *SSC* side scatter, *FSC* forward scatter

A series of optimization steps was carried out prior to analysis and sorting using the MOFLO Astrios EQ and the Kaluza 1.3 software package (both Beckman Coulter, Copenhagen, Denmark). The Sphero Ultra Rainbow Single Peak Fluorescent Particles (Spherotech, Salt Lake City, USA) were used for the optimization of stream alignment and quality control (QC). Furthermore, a battery of beads was used to ensure that the flow cytometer was fine-tuned to discriminate between noise and dimly stained cells and that each channel was set within its linear range of signal amplification. This was especially important, since the flow cytometer used represents a stream-in-air design with manual calibration of the stream. Consequently, the Sphero Ultra Rainbow Six Peak Fluorescent Particles (Spherotech) were used to determine signal-to-noise ratio (S/N), the linear range and the coefficient of variation (CV) at a range of voltage settings for each of the photomultiplier tubes (PMTs) (Fig. 2a and b) as described previously [36], thus providing basis for optimization of PMT voltages for each of the specific fluorochromes utilized in the present setup. The PMT voltages were determined with the aid of the BD CompBeads Anti-Mouse Ig, κ (BD Biosciences) conjugated to our antibody arsenal. The settings providing the highest S/N ratio without the occurrence of double negativity were used (Fig. 2c) and the gains were adjusted so an unstained sample produced a bell-shaped emission with a median fluorescence of 10^1^. Great care was also taken to ensure reproducibility between the experiments. To achieve this, the Sphero Ultra Rainbow Single Peak Fluorescent Particles were run along each sample to confirm the uniformity of the CV and the geometric mean of the fluorescence intensity (GMFI) (Fig. 2d). Acceptable flow cytometer alignment was defined as a successful automatic quality control combined with the CVs and GMFIs varying less than 4.5% and PMT voltages being within the linear range. Finally, compensation values were established for each run to control for the bleed-through utilizing the BD CompBeads Set Anti-Mouse Ig, κ and the AutoComp Wizard in Summit 6.1 (Beckman Coulter) and the Kaluza 1.3 when analyzing data. Additional manual fine-tuning of the compensation matrix was done whenever necessary.

To validate the cells for analysis and sorting, four gates to exclude flow cytometer instability (Fig. 2e), noise and cell debris (Fig. 2f), cell doublets (Fig. 2g), and dead cells (Fig. 2h) were invoked. Based on this strategy, the cutoff value for the positivity was defined as the top 2.5 percentile of the FMO control for a given marker. Added measures were taken to preserve the viability and yield of the cells from the sorting procedure. To this end, the flow-line was cleaned by using the Moflo FACS Cleaner (Beckman Coulter) and 70% ethanol, and the collecting tube holder was cooled to 5 °C. Moreover, the dissociation of cells was aided with the Accumax Solution (Sigma-Aldrich, Brøndby, Denmark) and straining through a 35-μm cell strainer (BD Biosciences). The sorting was performed with the sample chamber and the sheath fluid tank pressures being 25.3 and 25 psi, respectively, and the voltage at the deflection plates was set at 3800 V. Stable deflection beams were optimized through careful setting of charge phase and defanning. The cells were sorted in a sterile sheath fluid into the growth medium containing 20% FCS and 0.25 mM HEPES.

### CFU assay

The frequency of colony-forming units (CFUs) was determined directly in the sorted populations and in the populations after each of the ensuing passages by limiting dilution [37]. In brief, the cells were seeded in quadruplicates in densities ranging from 1 to 30 cells per well, and after 14 days, the cultures were fixed with 4% formaldehyde (AppliChem, Esbjerg, Denmark) and stained using 0.05% crystal violet (Sigma-Aldrich). Wells containing one or more colonies were noted and the proportion of CFUs calculated using L-Calc Software (Stem Cell Technologies, Vancouver, Canada). Data not following the Poisson distribution were excluded from analysis.

### Endothelial scratch assay

Human dermal microvascular endothelial cells (PromoCell, Heidelberg, Germany) were seeded in quadruplicates in a 96-well plate at 12,000 cells/cm^2^ in Endothelial Cell Growth Medium MV2 (MV2; PromoCell). After reaching confluency, the monolayers were scratched using the Wounding Pin Tool (V&P Scientific, Radway Green, United Kingdom), and the MV2 media was added as negative control, α-MEM supplemented with 10% FCS and antibiotics was added as vehicle control, and the conditioned media from P2 ASCs (C_FSC_) or sorted subpopulations (SP1–4) were added for testing the wound-healing properties. The conditioned media were based on α-MEM supplemented with 10% FCS and antibiotics and produced during a 3-day culture period prior to the end of P2. All FCS used in this study originated from the same batch and lot. The progress of damage reparation was monitored by time-lapse microscopy every 2 hours for a total of 12 hours using a Zeiss Axio Observer.Z1 microscope equipped with an AxioCam MRm camera and AxioVision software package (Carl Zeiss, Birkerød, Denmark). Relative wound size at each time point was analyzed using the TScratch software [38].

### Differentiation assays

ASCs were analyzed for their multilineage capacity by differentiating toward adipo-, osteo- and chondrogenic lineages using procedures as implemented in our laboratory [39–43]. The adipogenic differentiation was initiated in A-MEM with 10% FCS, 0.1 μM dexamethasone, 0.45 mM 3-Isobutyl-1-methylxanthine (IBMX), 0.17 μM insulin, and 0.2 mM indomethacin (all from Sigma-Aldrich). Lipid accumulation was visualized by staining with oil red O (Sigma-Aldrich). For osteogenic induction, the cells were grown in A-MEM supplemented with 10% FCS, 0.1 μM dexamethasone, 50 μM L-ascorbic acid, 0.05 μM calcitriciol, and 10 mM glycerol-2-phosphate (all from Sigma-Aldrich). Alizarin red (Sigma-Aldrich) was used to reveal the mineralization of extracellular matrix. The chondrogenesis was accomplished using micromass cultures maintained in Dulbecco’s Modified Eagle’s Medium supplemented with 25 mM glucose (Invitrogen), 0.4 nM TGF-β1 (Sigma-Aldrich), 100-fold diluted ITS-G (Gibco, Taastrup, Denmark), 0.35 μM L-proline, 0.17 μM L-ascorbic acid 2-phosphate, and 0.1 μM dexamethasone (all from Sigma-Aldrich). The extent of differentiation was assessed from thin paraffin sections after staining with alcian blue 8GX (Sigma-Aldrich).

### Real-time RT-PCR

The transcriptional activation of lineage-specific differentiation genes, including PPAR-γ2, Sox9, and Osteocalcin, and the reference genes, including cyclophilin A (PPIA) and tyrosine 3/tryptophan 5-monooxygenase activation protein (YWHAZ), was carried out as described previously [16, 44]. Briefly, total RNA was isolated using the Aurum Total RNA Mini Kit (Bio-Rad, Copenhagen, Denmark) and the purity and concentration were determined spectrophotometrically using the Agilent RNA 6000 Nano kit (Agilent Technologies, Waldbronn, Germany). The iScript cDNA synthesis kit (Bio-Rad) was used to carry out reverse transcription. The amplification reactions were performed on a CFX Connect Real-Time PCR Detection System (Bio-Rad) in a final volume of 20 μl containing 5 pmol of each primer (DNA Technology, (Aarhus, Denmark) (Additional file 1: Table S1) and 10 μl cDNA using SYBR Green PCR Supermix (Bio-Rad). The thermal cycling protocol involved initial denaturation at 95 °C for 3 min and was followed by 40 cycles of denaturation at 95 °C for 10 sec and primer annealing and elongation for 30 sec at predetermined optimal primer-specific temperatures. To test for the specificity of the product, a melt curve function of the CFX Manager software (Bio-Rad) was invoked. A standard curve derived from the pool of all cDNA samples was used to calculate relative expression of each gene.

### Statistical analysis

The data were derived from two identical, independent experiments each with replicates, and technical replicates, whenever applicable, and they are presented as mean + standard error of the mean (SEM). Prior to statistical analysis using one-way ANOVA, the normality of distribution and equality variances were tested with the Shapiro-Wilk and Equal Variance Tests in SigmaPlot 12.0, respectively (Systat Software, San Jose, CA, USA). Statistical significance was assigned to *p* values < 0.05.

## Results

### Evolution of the subpopulation phenotype

**Fig. 3:**
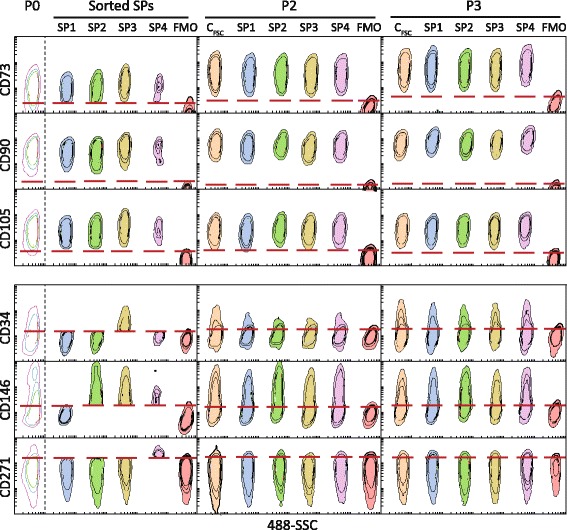
Evolution of immunophenotype after sorting according to specific co-expression patterns. The subpopulations with different combinations of six CD markers (SP1–4) and the control population sorted on the forward scatter (C_FSC_) were cultured and analyzed at the end of the next two passages (P2 and P3). The expression levels are plotted against the 488-SSC as contour plots to visualize progression in expression patterns. P0 represents analysis of the cell suspension just prior to sorting, and the *red lines* represent cutoff values for sorting gates as determined from FMOs. *Abbreviations*: *FMO* fluorescence minus one

The cells expanded during P0 were sorted into four subpopulations (SP1–4) based on the co-expression of three major epitopes CD73, CD90, and CD105, and four different combinations of minor markers, including CD34^−^CD146^−^CD271^−^, CD34^−^CD146^+^CD271^−^, CD34^+^ CD146^+^CD271^−^, and CD34^−^CD146^+^CD271^+^ (Fig. 3). Additionally, a population was included (C_FSC_), which was sorted on a simple FSC/SSC gate in order to control for the potential impact the sorting procedure might introduce into the experiment.

The expression patterns were investigated at the time of sorting (P0) and for the subpopulations at passages P2 and P3 (Fig. 1). With respect to major markers, all of the cells at P0 expressed CD90 and CD105, in contrast to CD73, which was expressed on 96.3% of cells. As a general paradigm regarding the CD73 and CD90, the level of expression increased in the course of culturing, as indicated by an increase in the GMFI. Thus, the CD73 featured a 1.5-fold upregulation at P3 relative to the initial phenotype, while the CD90 was upregulated 1.7 times. At P3 cells in all subpopulations co-expressed the three major markers. As for the minor markers, most of the cells at P0 were negative for CD34 and CD271, leaving thus 24.3% and 3.8% of cells positive, respectively, while a majority of the cells were positive for the CD146 (31.5%). It is interesting that upon culturing, the expression pattern of all minor epitopes became highly reminiscent of that of the C_FSC_, irrespective of whether the epitope was selected in a given subpopulation or not. This evolution, however, occurred more rapidly for CD146 and CD271, where it appeared already at P2, whereas, for CD34 it was delayed until P3.

**Fig. 4:**
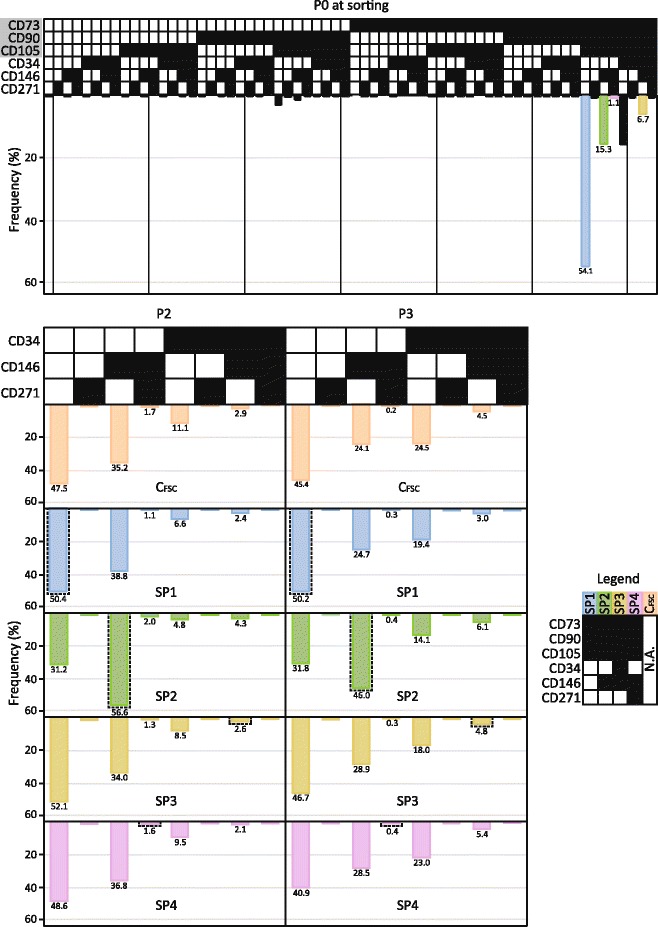
Frequency of repertoires based on co-expression of six selected CD markers as a function of in vitro expansion. Sorted subpopulations SP1–4 and the control population C_FSC_ sorted on the forward scatter were analyzed at the end of passages two and three (P2, P3). All possible repertoires are shown at P0. Only co-expression variants for CD34, CD146, and CD271 are shown at P2 and P3, since the CD73, CD90, and CD105 markers were invariably co-expressed. The shaded area at P0 at sorting denotes profiles associated with ASC hallmark markers, and the frequencies are also highlighted with individual bars. The legend indicates full profiles of the originally sorted repertoires, and these are highlighted in the plots by a *stippled line*. The data are presented as means from two independent experiments. ■ The marker is expressed. □ The marker is not expressed. *Abbreviations*: *N.A*. not applicable

When considering the surface repertoire at P0, it is significant that four distinct combinations of markers constituted more than 90% of the whole population (Fig. 4). The largest subpopulation (54.1%) featured only the major epitopes, whereas the three smaller subpopulations co-expressed in addition single markers CD34 (15.7%) or CD146 (15.3%), or combination thereof (6.7%). In the current investigation, we opted to follow a minor subpopulation co-expressing the CD146 and CD271 (1.1%) rather than the more abundant CD34+ subpopulation based both on the literature and our own data [45, 46]. Upon in vitro expansion, the repertoire within the C_FSC_ cultures (C_FCS_ at P2) redistributed and stabilized in a manner that the cells co-expressing exclusively the major markers remained a dominant although slightly smaller subpopulation (47.5%), the CD146+ subpopulation became the second most abundant (35.3%), the subpopulations expressing CD34 alone or in combination with CD146 decreased to 11.1% and 2.9%, respectively, and the CD146 and CD271 double-positive cells remained to represent only a small proportion of 1.7%. Interestingly, all sorted subpopulations re-expressed the missing epitopes so as to assume the generic paradigm of C_FSC_ cells, irrespective of the duration of culture. Slightly differently behaved the cells sorted for the expression of CD146 (SP2), where this phenotype remained the most prevalent one throughout the culturing (P2, 56.6%; P3, 46.0%).

### Subpopulation stem cell characteristics

**Fig. 5:**
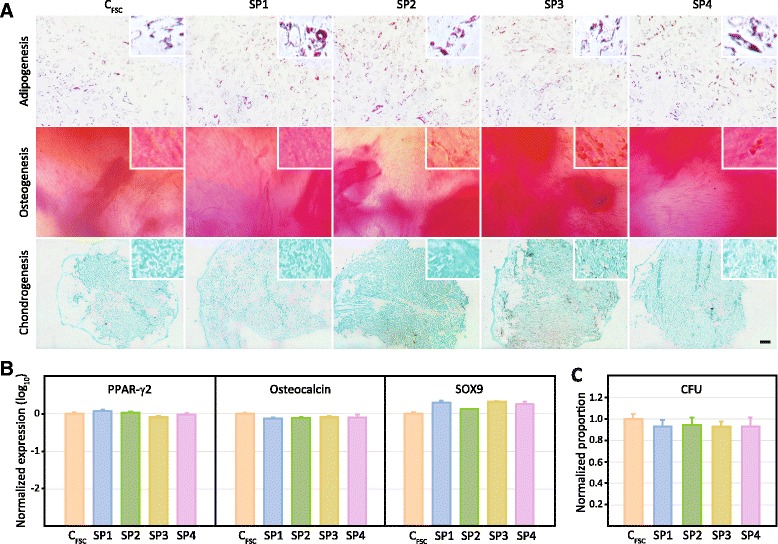
Stem cell properties of the phenotypically selected subpopulations SP1–4 and the control subpopulation C_FSC_ sorted on the forward scatter. **a** Histochemical analysis of the trilineage differentiation using oil red O, alizarin red, and alcian blue for adipo-, osteo-, and chondrogenesis, respectively. The insets depict fourfold zoom from the large field micrographs. The scale bar denotes 100 μm. **b** Transcriptional expression of differentiation regulators by real-time RT-PCR from two independent experiments carried out in triplicate (n = 6). **c** The colony-forming capacity was assessed after staining with crystal violet from two independent experiments carried out in replicate (n = 16). The data are presented as mean + SEM

The trilineage differentiation potential of the C_FSC_ and SP1–4 cells was tested at P2 by subjecting the cultures to adipo-, osteo-, and chondrogenic regimens (Fig. 5). The qualitative assessment by bright-field microscopy demonstrated a comparable extent of differentiation irrespective of the phenotype (Fig. 5a). This was further corroborated by real-time RT-PCR, where no significant difference in the expression of major differentiation markers could be found among the discrete subpopulations (Fig. 5b). The similarity between the subpopulations was at the same stage of culture also confirmed by the CFU analysis, which could not reveal any significant differences in the efficiency of maintenance of the precursor cells (Fig. 5c).

### Subpopulation wound-healing capacity

**Fig. 6:**
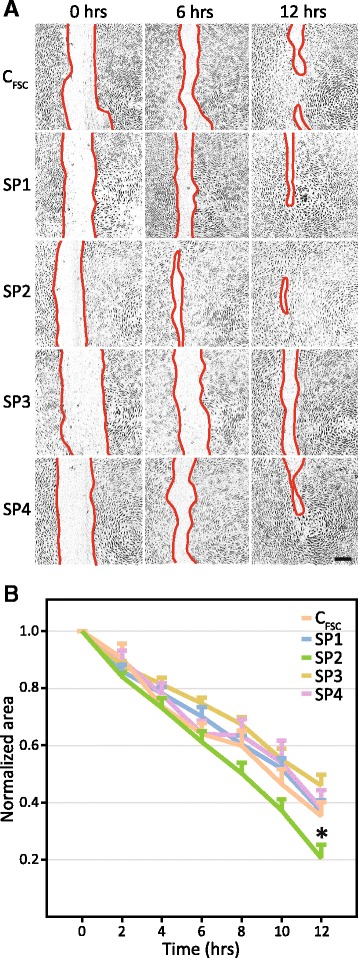
Modulation of endothelial wound healing by conditioned media derived from the phenotypically selected subpopulations SP1–4 and the control subpopulation C_FSC_ sorted on the forward scatter. **a** Representative micrographs from the scratch assay. Cell-free surface is demarcated by a *red line*. The scale bar denotes 200 μm. **b** Quantitative assessment of the wound healing from two independent experiments carried out in replicate (n = 12). The data are presented as mean + SEM. ^*^Statistically different from C_FSC_ and all other SPs at *p* < 0.05

The capacity of the C_FSC_ and SP1–4 cells to promote wound healing was examined at P2 using a scratch assay (Fig. 6 and Additional file 2: Figure S1). The phase-contrast microscopy indicated a differential rate of wound closure upon stimulation with the ASC conditioned media, with SP2 supernatant being apparently the most efficient (Fig. 6a). The kinetic analysis of the monolayer response to different media demonstrated the superiority of SP2 throughout the assay period, and a statistically significant effect could be confirmed after 12 hours (Fig. 6b).

## Discussion

The ASCs as well as other mesenchymal stem cells derived from numerous tissue types were shown to express in vivo and in vitro a gamut of surface markers, the specific combinations of which may ultimately define distinct repertoires [26, 31, 33, 46–50]. Previous studies suggested that some of the markers may play a role in the ASC functionality, such as angiogenesis, differentiation, migration, and secretome [1, 27, 50–52]. These approaches were based on the selection of single surface markers and characterization directly after sorting, giving thus a limited emphasis to the co-expression patterns [29, 31, 45, 48, 53–55]. In continuation of these efforts we sought to obtain insight into how more complex surface marker variants are maintained during culturing and whether they have a potential to provide for progeny populations with enhanced specific functionality. Such study would further our understanding of the significance of different combinations of more and less frequent markers that specify the discrete subpopulations. It is plausible that overrepresentation of unique repertoires in certain donors would provide at least partial explanation for great inter-individual differences in particular regenerative applications. Consequently, specific donors or subpopulations could be rationally selected to provide for optimal clinical response.

Based on previous investigations [14, 15, 20–23], we identified CD73, CD90, CD105, CD34, CD146, and CD271 as six surface markers with major relevance for determination of ASCs. In this study, not all combinations appear equally permissible, but 95% of early ASCs (P0) are found in the compartment defined by CD73^+^90^+^105^+^ phenotype. Of these cells, 54% does not express any of the other studied markers, 16% co-express CD34, 15% co-express CD146, and 7% co-express both of them. To study the effect of in vitro culture on the properties of discrete subpopulations, we opted to include a minor SP4^CD146+271+^ in favor of the sole CD34^+^ variant since our previous investigation indicated frequent occurrence of this phenotype, ranging from 5 to 20% [46]. Low frequency of the minor combination CD146^+^CD271^+^ in the current study underscores significance of factors underlying interindividual variability, in this case most likely those related to age [47].

Interestingly, our findings show that in the progeny population stemming from the single sorted profile, a distribution of phenotypic variants reappears so as to resemble to original pre-sorted population (P0). Depending on the originally selected markers, all downstream lines thus as a result of culturing gained, lost, or gained and lost one or more markers, ultimately resembling each other. This indicates that a restricted heterogeneity rendered by distinct physiologically important repertoires is critical for stability and viability of ASC cultures, and further raises the question about possible mechanisms involved in the mutual intervariant stimulatory interactions enabling survival in vitro. Importantly, despite obvious resemblance, the downstream lines may display differential functionality under appropriate conditions. Selection for the CD73^+^CD90^+^CD105^+^CD34^−^146^+^271^−^ phenotype (SP2) clearly resulted in a line with increased endothelial stimulation. Given the scope of epitopes selected in this study, it seems that of the minor markers, the CD146 is necessary and sufficient to provide for augmented paracrine support of endothelial cells. An explanation for this functional significance of the CD146 probably stems from the fact that it has been identified as a cell adhesion molecule and a hallmark epitope of endothelium regulators, the pericytes. Pericytes are perivascular cells that have been located perivascularly in almost all tissues [29], and in addition to a major role in angiogenesis through an interplay with endothelial cells [21, 28, 56–58], they have been found to influence homeostasis through contraction of blood vessels [21], or regulate coagulation and lymphocyte activation and migration [59]. Nevertheless, to confirm the existence of true pericyte phenotype, the presence of additional markers, such as the α-SMA, NG2, and PDGF-B receptor and absence of CD31, CD34, CD45, and CD56 would have to be confirmed [21, 27–30, 57].

There is no obvious explanation why selection of SP2 repertoire would produce such functional outcome when all other progeny lines also contained this variant in a substantial proportion. The level of CD146 expression could be of importance, as was suggested previously by some investigators [31, 33], but that such simple quantitative indicator plays a role does not seem to be supported by our data. The answer thus may reside in differences between relative proportions of individual repertoires within the progeny lines or in the way how CD146 expressed in the context of other epitopes defines the selected parental population. The issue of more defined subpopulations appears intricate, since some previous data contradict our findings by highlighting the CD34^+^CD146^+^ phenotype as highly pro-angiogenic [45]. Clearly, further studies are necessary to shed more light on the biological significance of complex repertoires, especially in terms of molecular events involved in the secretion of paracrine factors. By the same token, it would be of interest to determine how significant is the association of CD146 with the regenerative potential in the clinical setting. Since, as mentioned above, this phenotype plays a role in revascularization processes, it can be envisaged that in conjunction with appropriate preconditioning regimen, such as hypoxia [8, 15, 16, 60–63], substantially improved therapeutic outcome would be achieved.

## Conclusions

Downstream lines from ASCs selected on the basis of specific surface repertoires have a tendency to assume upon culturing a phenotypical distribution reminiscent of the original population. Depending on the originally selected markers, all lines thus as a result of culturing gained, lost, or gained and lost one or more markers, ultimately resembling each other. Although general assays for stemness, such as the trilineage differentiation and CFU capacity, could not reveal any difference between the lines, specific functional analysis based on the endothelial model of wound healing demonstrated superiority of one of the propagated subpopulations. This property appeared to be rendered by selecting for the CD73^+^CD90^+^CD105^+^CD34^−^CD146^+^CD271^−^ phenotype. The sole expression of CD146 on the background of the CD73, CD90, and CD105 seems to be critical, since co-expression of additional minor marker, such as CD34 or CD271 abrogated the functional advantage. Based on this example, it is possible that other discrete subpopulations may provide for progeny populations that would have the potential to deliver in a specific clinical scenario an effect superior to that of crude ASCs.

## Additional files

Additional file 1: Table S1. Primer sequences and annealing temperatures. (DOCX 29 kb)
Additional file 2: Figure S1. Modulation of endothelial wound healing by conditioned media derived from the phenotypically selected subpopulations SP2 or the control Endothelial Cell Growth MV2 and media. (A) Representative micrographs from the scratch assay. Cell-free surface is demarcated by a *red line*. The scale bar denotes 200 μm. (B) Quantitative assessment of the wound healing from two independent experiments carried out in replicates (n = 12). The data are presented as mean + SEM. ^*^Statistically different from MV2 and α-MEM at *p* < 0.05. (EPS 105730 kb)

## Abbreviations

AGM: average geometric mean
ASC: adipose-derived stem cells
C_FSC_: control subpopulation sorted on FSC/SSC gate
CFU: colony-forming unit
CV: coefficient of variation
FACS: flow cytometry-associated cell sorting
FMO: fluorescence minus one
FSC: forward scatter
GMFI: geometric mean of fluorescence intensity
IBMX: 3-Isobutyl-1-methylxanthine
PMT: photo multiplier tube
P*n*: passage, where *n* denotes the number
PPIA: cyclophilin A
S/N: signal-to-noise ratio
SEM: standard error of the mean
SP: subpopulation
SSC: side scatter
SVF: stromal vascular fraction
YWHAZ: tyrosine 3/tryptophan 5-monooxygenase activation protein
