# Supplementary material for: Discrete adipose-derived stem cell subpopulations may display differential functionality after in vitro expansion despite convergence to a common phenotype distribution
Source: Stem Cell Res Ther. 2016 Dec 1;7:177. doi: 10.1186/s13287-016-0435-8 (PMC5134234; doi:10.1186/s13287-016-0435-8)
Supplement: Additional file 1: Table S1. — Primer sequences and annealing temperatures. (DOCX 29 kb) [file 13287_2016_435_MOESM1_ESM.docx]

Additional file 1: Table S1. Primer sequences and annealing temperatures

| Target | Forward Primer Sequence | Annealing temp (°C) | |  |
| --- | --- | --- | --- | --- |
| PPAR-γ2 | F 5’- TCA GGT TTG GGC GGA TGC -3’  R 5’- TCA GCG GGA AGG ACT TTA TGT ATG -3’ | | 68 |  |
| Osteocalcin | F 5’- GAG CCC CAG TCC CCT ACC C -3’  R 5’- GCC TCC TGA AAG CCG ATG TG-3’ | | 65 | |
| SOX9 | F 5’- TTC GGT TAT TTT TAG GAT CAT CTC G -3’  R 5’- CAC ACA GCT CAC TCG ACG ACC TTG -3 | | 60 | |
| PPIA | F 5’- TCC TGG CAT CTT GTC CAT G -3’  R 5’- CCA TCC AAC CAC TCA GTC TTG -3’ | | 60 | |
| YWHAZ | F 5’- ACT TTT GGT ACA TTG TGG CTT CAA -3’  R 5’- CCG CCA GGA CAA ACC AGT AT -3’ | | 60 | |

F, forward primer; R, reverse primer
